# Bioinformatics analysis to explore the potential prognostic utility of hsa-miR-103a-3p in head and neck squamous cell carcinoma

**DOI:** 10.1038/s41598-025-26188-6

**Published:** 2025-11-21

**Authors:** Hanin Shoukry, Aliaa A. Elsherbiny, Sally A. Fahim

**Affiliations:** 1 School of Pharmacy, New Giza University (NGU), Newgiza University (NGU), km 22 Cairo-Alexandria Desert Road, P.O. Box 12577, Giza, Egypt; 2 Department of Biochemistry, School of Pharmacy, Newgiza University (NGU), Newgiza, km 22 Cairo-Alexandria Desert Road, P.O. Box 12577, Giza, Egypt

**Keywords:** HNSCC, hsa-miR-103a-3p, Bioinformatics, Target genes, Gene ontology, Molecular biology, Genetics, Gene expression

## Abstract

Head and neck squamous cell carcinoma (HNSCC), the seventh most common cancer globally, has not yet seen an exploration of the specific role of hsa-miR-103a-3p. This study employs integrated bioinformatics analysis to investigate the function, regulatory patterns, and prognostic significance of hsa-miR-103a-3p in HNSCC. The Kaplan-Meier database was used for pan-cancer prognostic analysis of hsa-miR-103a-3p. Expression significance was assessed using the dbDEMC and GSE124566 dataset from GEO. TargetScan, RNA22, miRDB, and miRWalk identified potential targets, while GEPIA 2.0 provided differentially expressed genes (DEGs). Functional and pathway enrichment analyses were conducted using ShinyGO and KOBAS. Elevated hsa-miR-103a-3p expression correlated with poor overall survival and was upregulated in HNSCC tissues. Integration of four target prediction tools yielded 297 candidate genes. Among them, *HOXD10*, *HMGA2*, and *THY1* showed the highest expression, whereas *UBL3*, *AMOT*, and *PDK4* displayed low levels. *UBE2Q1*, *ZFPM2*, and *ATXN1* demonstrated the strongest accessibility to hsa-miR-103a-3p binding. Network analysis revealed interactions with transcription factors and tumor-related genes, while enrichment highlighted involvement in upstream regulators and downstream cancer-associated pathways. This study identified 297 target genes and clarified the regulatory role of hsa-miR-103a-3p through interaction networks, transcription factor associations, and pathway enrichment. These findings suggest a potential oncogenic role for hsa-miR-103a-3p in modulating the cAMP signaling pathway, proteoglycans in cancer, and related mechanisms, thereby linking its molecular regulation to poor prognosis and potential therapeutic targeting in HNSCC.

**Supplementary Information:**

The online version contains supplementary material available at 10.1038/s41598-025-26188-6.

## Introduction

Cancer is a serious health concern, and a primary contributor to mortality. Even with the implementation of modern screening technologies and the continual advancements in treatments, cancer survivors still experience physical and psychological issues^1^. By the end of 2024, there are projected to be around 28.9 million new cases of cancer worldwide, resulting in 16.2 deaths each year^2^. As per the national cancer registry programme (NCRP), cancer incidence rate in Egypt was 166.6 per 100,000 in both sexes, which represents a three-fold increase compared to 2013. This number represents a tremendous burden on society; thus, the development of a new diagnostic tool with the improvement in accuracy is crucial to enhance the survival^3^.

Head and neck cancers (HNCs) encompasses a wide range of complex malignancies, primarily originating from the epithelial cells of areas such as the oral cavity, larynx, pharynx, paranasal sinuses, nasal cavities, and salivary glands^4^. 90% of all HNCs are known as squamous cell carcinoma (HNSCC) and originate from the mucosal lining in these regions. Additionally, HNSCC is a diverse condition and ranks as the seventh most prevalent cancer worldwide with 890,000 reported incidences and 450,000 deaths^5^. The occurrence of HNSCC is steadily increasing and is expected to surge by 30% by 2030, as reported by the Global Cancer Observatory^6^. The risk of developing these cancers is closely linked to individuals who are susceptible to prolonged sun exposure, chew areca nuts, consume excessive amounts of alcohol and/or tobacco, have genetic susceptibility, or get infected with human papillomavirus (HPV)^7^. Because of their aggressive nature and genetic complexity, HNSCC remain challenging, and make them prone to resistance despite the innovations in targeted therapies, surgery, and radiotherapy^8,9^. Early detection of cancer enhances the chances of successful treatment, minimizing its impact on surrounding tissues and organs. The high morbidity and mortality rates associated with HNSCC underscore the need for prompt and precise diagnosis.

Hence, the search for a novel biomarker is crucial. Non-coding RNAs (ncRNAs) offer a diagnostic advantage due to their robust stability in various biological fluids, tissue-specific expression patterns, strong associations with diseases and the ease of quantification at low concentrations, which underscores their accuracy in diagnosis^10^. NcRNAs are a diverse and heterogeneous set of molecules that can vary in length, with long non-coding RNAs (lncRNAs, > 200 nucleotides) and short non-coding RNAs (200 nucleotides) being sub-classified into MicroRNAs (miRNAs), short interfering RNAs (siRNAs), piwi-interacting RNAs (piRNAs), and small nucleolar RNAs (snoRNAs)^11^. Within these, in depth, miRNAs are a group of evolutionary small, single-stranded, highly conserved non-coding RNAs comprised of 19–24 nucleotides^12^. Through transcription by RNA polymerases II and III, pre-miRNAs are produced and undergo a series of cleavage events to generate a mature miRNA^13^. To shut down the expression of a gene, miRNAs interact with their complementary mRNA, where the degree and nature of homology between the two molecules dictates the gene silencing mechanism. These tiny molecules have various roles in biological processes such as cell division, differentiation, apoptosis, and metabolism^14^. Maladjusted miRNAs can play a significant role in the development of cancer. There are various types of miRNAs, each with its own unique way of modulating gene expression, whether as tumor suppressors or oncogenes. In cancer, these miRNAs can bypass growth inhibitors, sustain proliferative signals, prevent cell death, induce angiogenesis, and initiate invasion and metastasis^15^. Furthermore, miRNAs hold great promise as biomarkers for the diagnosis, prognosis, and prediction of cancer^16^. They can be detected in cancerous tissue as well as bodily fluids, making them valuable tools in the clinical management of cancer patients^17^ Numerous research studies have confirmed miRNAs’ value. As a result, miRNAs appear as a reliable and promising tool for future applications, allowing for the development of more targeted and precise therapeutic interventions^17–19^.

Hsa-miR-103a-3p has been discovered to have an important role in the regulation of carcinogenesis. Consequently, the dysregulation of hsa-miR-103a-3p has shown a dual effect on cancer by functioning in some types as an oncogene; in others, it can serve as a proto-oncogene. In a subset of research, it is reported that hsa-miR-103a-3p is an oncomiR in several cancer types, including cervical cancer^20^, breast cancer^21^, non-small cell lung cancer^22^, colorectal cancer^23^, gastric cancer^24,25^, endometrial cancer^26^. Contrarily, the expression of hsa-miR-103a-3p was known to be down-regulated in prostate cancer^27^ and hepatocellular carcinoma^28^. Overexpression of miR-103 suppresses *MMP-9* and *MMP-2* by downregulating *SALL4*, thereby reducing the proliferation and invasive capacity of oral squamous cell carcinoma Tca8113 cells^29^. The expression of miR-103a has been shown to possess independent predictive and prognostic significance in cancer patients^30,31^, underscoring its importance in cancer biology through the modulation of multiple signaling pathways^32^. Despite this, its specific role, target pathways, and mechanistic contributions in cancer, HNSCC remains largely unexplored. To our knowledge, this is the first study employing an integrative bioinformatics approach to investigate the potential prognostic utility of hsa-miR-103a-3p in HNSCC, providing novel insights into its biological significance and potential as a biomarker in this cancer type.

Therefore, the identification and thorough functional analysis of target genes regulated by hsa-miR-103a-3p might provide further insight into the mechanism underlying the development of HNSCC. Within this study, we conducted a series of in-silico investigations with the goal of elucidating the fundamental roles and regulatory patterns of hsa-miR-103a-3p.

## Methodology

### Screening of hsa-miR-103a-3p expression using DbDEMC database

The updated dbDEMC v3.0 (https://www.biosino.org/dbDEMC/index, accessed on October 7, 2023)^33^, an integrated database analyzes miRNA expression patterns across 40 cancer types using large-scale profiling data from GEO and TCGA, was used to examine the dysregulated expression of hsa-miR-103a-3p in tissue samples of cancer patients versus healthy individuals.

### Assessing the significance of hsa-miR-103a-3p expression through analysis of GEO database

The identification number (GSE124566) was searched for and sourced from the GEO database (https://www.ncbi.nlm.nih.gov/geo/, accessed on October 25, 2023)^34,35^. The GSE124566 dataset was conducted utilizing the GPL18402 platform (Agilent-046064 Unrestricted_Human_miRNA_V19.0_Microarray) and comprised a total of 20 samples. Additionally, the GEO2R software automatically computed the P-values for the data in GEO datasets. Within this dataset, the expression of hsa-miR-103a-3p was compared to samples of normal tongue tissue and tongue squamous cell carcinoma. This analysis involved the creation of both a scatter plot using Graphpad prism v9.5.1 and a volcano plot to visualize the differences in expression levels between these sample types.

#### hsa-miR-103a-3p correlation with overall survival in Pan-cancer using Km plotter

As an estimator method for the prognostic function, Kaplan-Meier, an integrated online global bioinformatics tool, was used to asses and graph survival probabilities as a function of time (https://kmplot.com/analysis/index.php?p=service, accessed on September 27, 2023)^36^. Pan-cancer overall survival analysis was performed to visualize the significance of hsa-miR-103a-3p expression. Based on the median expression value of hsa-miR-103a-3p, patient samples (*n* = 7642) with various cancer types were divided into abundant and low expression groups. The default parameters were employed in the present study, and the log-rank P value and median survival were calculated for the chosen cancer graph. False discovery rate (FDR) using the Benjamini-Hochberg method is computed to correct multiple hypothesis testing. The cutoff value with the highest significance (lowest FDR) is determined. In case of multiple cutoff values with identical significance, the cutoff with the highest hazard (HR) rate is selected for the final analysis.

#### Prediction of target genes for hsa-miR-103a-3p in HNSCC

To investigate the oncogenic effect of the elevated hsa-miR-103a-3p in the development of HNSCC, it was essential to utilize online integrated tools for predicting target genes. In the current analysis, putative target genes for hsa-miR-103a-3p were attained using the following algorithmic target prediction tools: TargetScan v8.0 database (https://www.targetscan.org/vert_80/, accessed on November 15, 2023)^37^, miRDB (https://mirdb.org/, accessed on November 15, 2023)^38^, RNA22 v2.0 (https://cm.jefferson.edu/rna22/Precomputed/, accessed on November 15, 2023)^39^, and miRwalk v3.0 (http://mirwalk.umm.uni-heidelberg.de/, accessed on November 15, 2023)^40^. Enhancing the reliability of our findings, we discovered the overlapping genes by employing a Venn Diagram through an online generator called Jvenn (https://xcmsonline.scripps.edu/lib/jvenn/example.html, accessed on November 19, 2023)^41^. Tumor/normal DEGs profile were identified through analysis of Gepia2 tool, and a log2 fold change < − 1 were considered down-regulated genes, while DEGs with a log2 fold change > 1.5 were exhibited up-regulated genes with a cutoff p-value < 0.05 (http://gepia2.cancer-pku.cn/#index, accessed on November 22, 2023)^42^. Additionally, miRNA-target accessibility data were acquired from the miRwalk database. These datasets were utilized for generating miRNA-gene network visualization figures through the application of Cytoscape software v3.10.1 ^44^.

### Understanding differentially expressed genes (DEGs) using gene ontology (GO) functional analysis and pathway enrichment analysis of HNSCC

In this study, the anticipated overlapping target list consisting of 297 gene symbols from four databases was subsequently employed to conduct functional annotation analysis using the ShinyGO database v0.77 (http://bioinformatics.sdstate.edu/go/, accessed on November 30, 2023)^44^. The obtained data from ShinyGO underwent a thorough verification process in the DAVID database. (https://david.ncifcrf.gov/tools.jsp, accessed on November 30, 2023)^45,46^ as well as KEGG pathway analysis was performed using the online analysis tool provided by KOBAS to investigate which DEGs is activated and suppressed in different class of pathways (http://bioinfo.org/kobas/genelist/, accessed on November 30, 2023)^47^. GO of the identified DEGs included several aspects: molecular function, cellular component, biological process, disease ontology, and pathways such as KEGG pathway and a significance level was set at a p-value < 0.05. Moreover, the projected targets of hsa-miR-103a-3p, as identified through miRWalk v3.0 (with a score exceeding 0.95), underwent separate GO annotation and KEGG pathway enrichment analysis. This strategic approach, which intended not to depend on the overlap of predicted targets, was implemented to prevent the missing of essential information. Finally, a bar chart created was employed to visually display the five selected sections to represent GO.

## Results

### Differential expression level of MiRNAs and high expression of hsa-miR-103a-3p

**Fig. 1:**
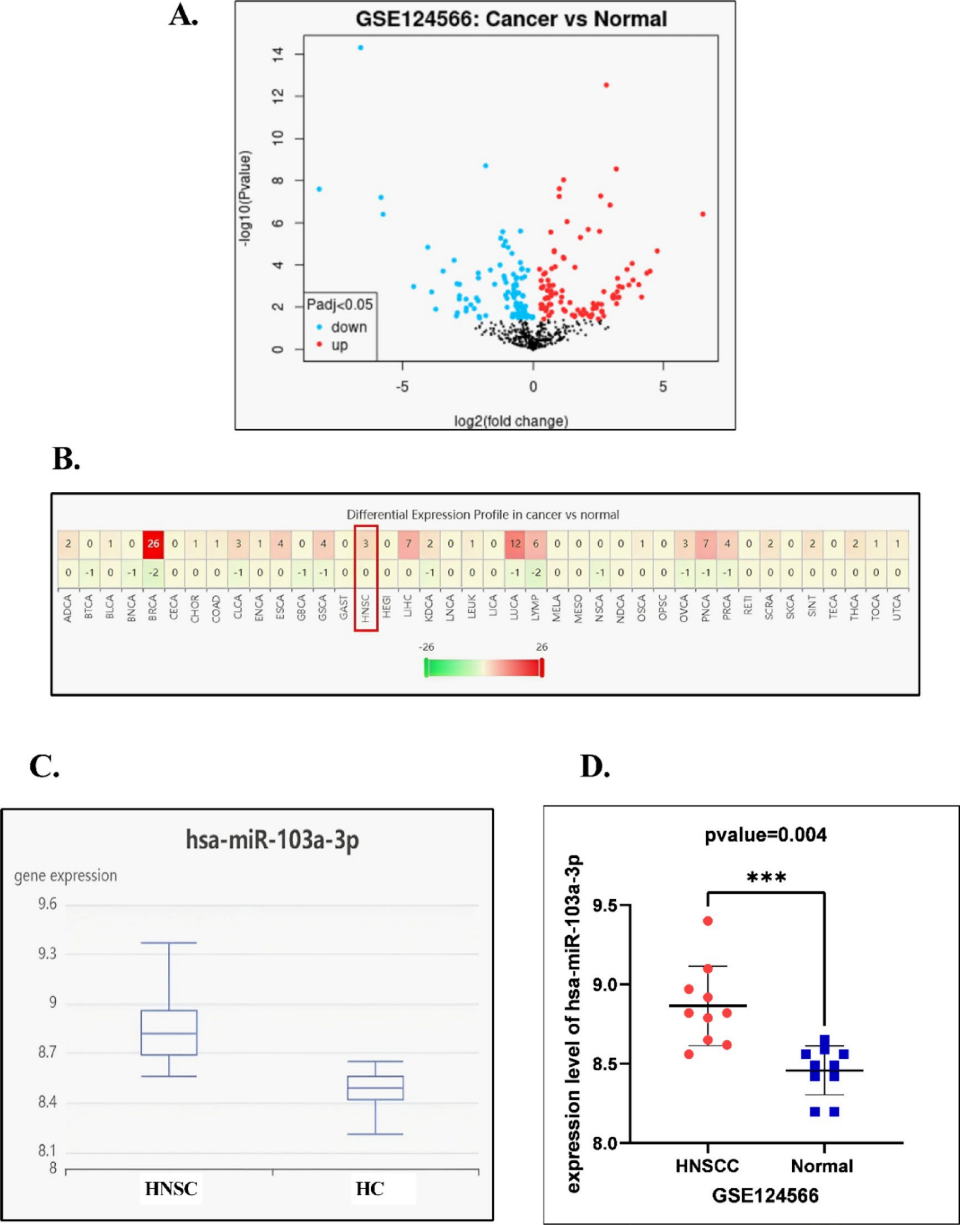
**(A)** Volcano map of differentially expressed miRNAs in HNSCC and normal squamous tissue obtained from GSE124566. **(B)** Heatmap calculated by dbDEMC software (red box represents hsa-miR-103a-3p expression in HNSCC). **(C)** Box Plot shows expression level median in HNSCC versus healthy controls (HC). (**D)** Analysis of hsa-miR-103a-3p expression in HNSCC and normal squamous tissue within GEO datasets.

The volcano plot comparing miRNA expression profiles between HNSCC tissues and normal squamous tissues (Fig. 1A) highlights a distinct set of significantly dysregulated miRNAs. Among these, several were markedly upregulated, represented by the red dots, with statistical thresholds set at |log2 fold change| > 0.4 and –log10(p-value) > 1.3 (*p* < 0.05). Notably, hsa-miR-103a-3p was significantly elevated, confirming its potential role in HNSCC progression with –log10(p-value) equals 3.5. This observation was further reinforced by the heatmap analysis (Fig. 1B), which demonstrated a clear clustering pattern differentiating cancerous from normal tissues. The consistently higher expression of hsa-miR-103a-3p in HNSCC samples compared to normal controls provides robust evidence supporting its involvement in the disease and justifies its selection for subsequent prognostic and pathway analyses. Furthermore, box plots illustrated that HNSCC tissues had a higher median (8.82 compared to 8.49) than adjacent normal tissues of healthy control (HC). The results demonstrated significant alterations in the regulation of hsa-miR-103a-3p with increased expression observed in cancerous tissue samples compared to healthy controls with log Fc = 0.41, and adj. p-value < 0.05 (p-value = 0.0131) as demonstrated in Fig. 1C.

A scatter plot was employed to depict the levels of hsa-miR-103a-3p in both HNSCC and normal tissue within the GSE dataset, consequently, a notable significance was observed with a logFC of 0.407, and the adjusted p-value = 0.0110 (Fig. 1D).

### In-silico prognostic analysis of hsa-miR-103a-3p using Km database

**Fig. 2:**
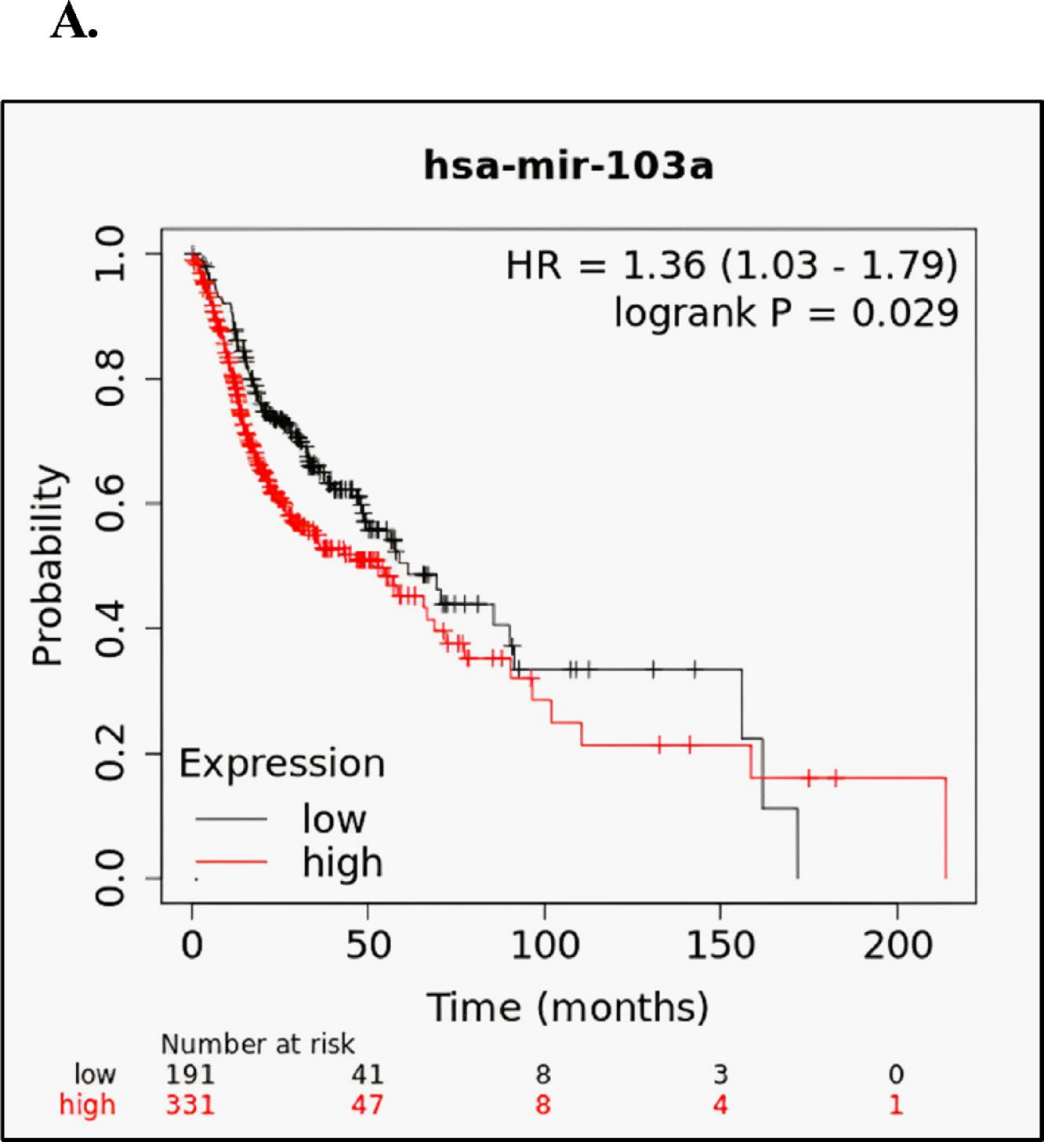
Survival curve for hsa-miR-103a-3p in patients with HNSCC Kaplan-meier database. High expression of hsa-miR-103a-3p (red) is significantly associated with poorer survival compared with low expression (black) (HR = 1.36, log-rank *P* = 0.029).

In the current research study, to figure out the prognostic value of hsa-miR-103a-3p, we conducted a survival analysis across multiple cancer types using the comprehensive Km dataset that covers various forms of cancer. From using the basic features of the Kaplan-Meier model, our results revealed significance (p-value < 0.05) with the following cancer types: Head-neck Squamous Cell Carcinoma (p-value = 0.029), Pancreatic Ductal Adenocarcinoma (p-value = 0.024), cervical squamous cell carcinoma (p-value = 0.042), and kidney renal clear cell carcinoma (p-value = 0.028) as represented in Figure S1. As remarkably the HNSCC has strong significance (p-value = 0.029) as well as notable difference in the overall survival rate between its low and high expression median of 12.4 months. Hence, the KM plotter results emphasized the prognostic significance of miR-103a-3p in the HNSCC (Fig. 2).

### Predicting integrated targets for hsa-miR-103a-3p

**Fig. 3:**
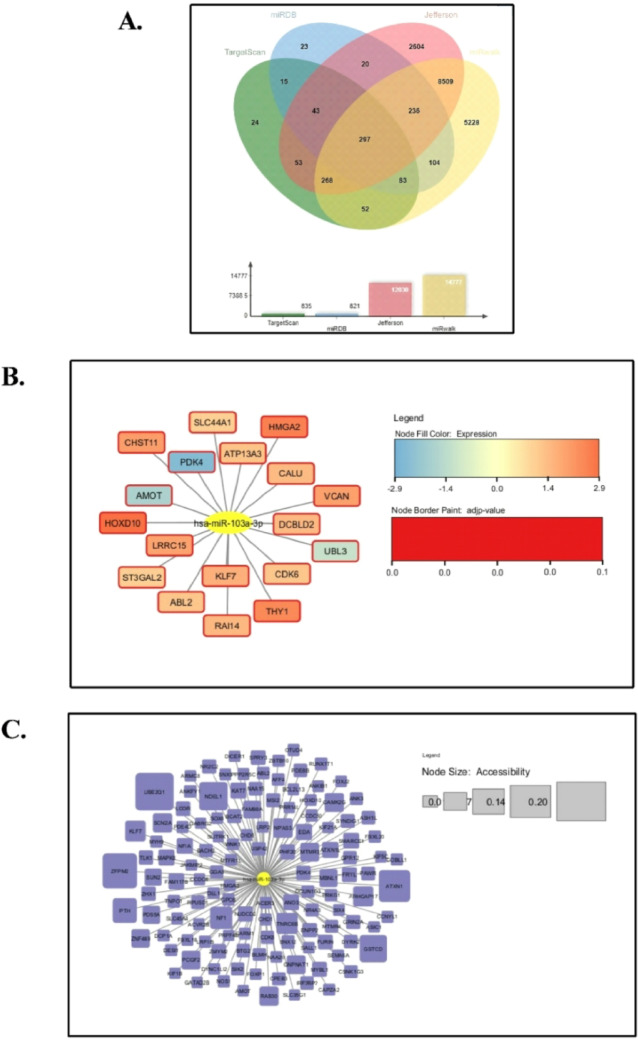
**(A)** Venn diagram displaying four potential miRNA-targets prediction tools: TargetScan v8.0, miRDB, RNA22 v2.0, and miRWalks v3.0 utilized to determine the targets of hsa-miR-103a-3p. **(B) (C)** The interaction network between hsa-miR-103a-3p and its targets was visualized using Cytoscape software v3.10.1. **(B)** The intensity of orange in the node corresponds to higher expression, with a red border indicating a significant p-value < 0.05. **(C)** Larger nodes represent increased accessibility for each gene.

The prospective hsa-miR-103a-3p target genes in HNSCC were estimated by using four target gene prediction platforms: TargetScan, miRDB, RNA22, and miRwalk. Using these three databases together increases prediction reliability by integrating sequence-based, machine-learning, and experimentally validated evidence, while reducing false positives compared to using a single database. Following that, the intersection of the forecasts from these four databases was examined. Our analysis revealed that 297 overlapping genes were identified as possible target genes for hsa-miR-103a-3p as illustrated in the Venn diagram (Fig. 3A). Out of these, Cytoscape software v3.10.1 was employed to visualize 18 genes expression profile selected from the mutual targets identified through the intersection analysis between the GEPIA2 target dataset and the 297 gene set. The visualization revealed that Homeobox D10 (*HOXD10*), High Mobility Group AT-Hook 2 (*HMGA2*), and Thy-1 cell surface antigen (*THY1*) exhibited the highest expression with log_2_FC = 2.851, 2.54, and 2.374 respectively (log_2_FC value higher than 1.5), On the contrary, Ubiquitin-like protein 3 (*UBL3*), Angiomotin (*AMOT*), and Pyruvate dehydrogenase kinase 4 (*PDK4*) revealed a low expression profile in HNSCC, with log_2_FC values of -1.211, -1.761, and − 2.549, respectively (log_2_FC value lower than 1). Additionally, an adjusted p-value < 0.05 in both high and low expression levels was set, confirming their robust association with HNSCC, as shown in Fig. 3B. The Cytoscape network figure was further utilized for a visualization involving 129 genes with an accessibility value cutoff of ≥ 0.006682259 obtained from the miRwalk database. The findings revealed that Ubiquitin-conjugating enzyme E2 Q1 (*UBE2Q1*), zinc finger protein, multitype 2 protein (*ZFPM2*), and Ataxin 1 (*ATXN1*) exposed the greatest accessibility to target sites for hsa-miR-103a-3p, as displayed in Fig. 3C.

### GO functional analysis of hsa-miR-103a-3p

**Fig. 4:**
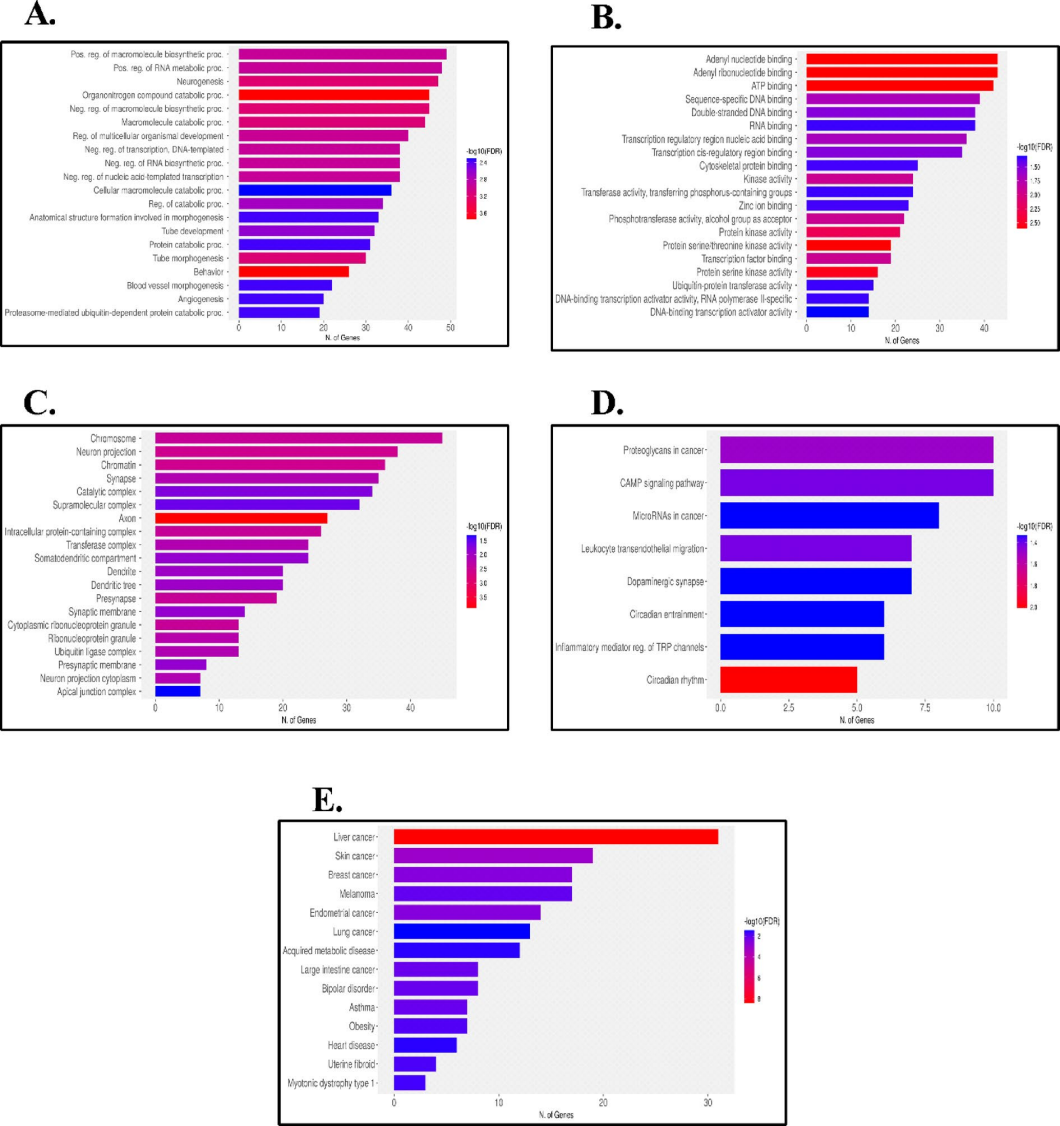
297 targets-based functional analysis, GO and KEGG pathway enrichment analysis. (**A)** Biological Processes, (**B)** Molecular Functions, (**C)** Cellular Components, (**D)** KEGG Pathways, (**E)** Disease Ontology.

In the present study, the application of GO and KEGG pathway were acquired to identify the most significantly enriched characteristic biological attributes and molecular pathways among DEGs with a significance threshold set at *p* < 0.05. The annotation results revealed that the identified DEGs are predominantly associated with processes such as the positive regulation of macromolecule biosynthetic processes, positive regulation of RNA metabolic processes, and the regulation of neurogenesis as illustrated in Fig. 4A. Regarding Molecular Functions, important processes include adenyl nucleotide binding, ATP binding, and adenyl ribonucleotide binding as presented in Fig. 4B. In terms of cellular components, key components involve the chromosome, neuron projection, and chromatin as exposed in Fig. 4C. Additionally, the KEGG pathway analysis showed enrichment in pathways related to proteoglycans in cancer, the cAMP signaling pathway, miRNAs in cancer, and leucocyte transendothelial migration as shown in Fig. 4D and FigureS2. Furthermore, Disease Ontology indicated enrichment in liver cancer, skin cancer, and breast cancer as presented in Fig. 4E. These enriched ontologies highlight their significance in comprehending the regulatory mechanisms that impact the behavior of cancer cells The most significant enriched terms (p-value < 0.05) within each GO category are displayed in Fig. 4. The figures depicting the results of the gene enrichment analysis for the miRwalk gene list can be found in FigureS3.

## Discussion

HNSCC stands as the seventh most widespread cancer across the globe, indicating its significant prevalence on a global scale^5^. MiRNAs serve an important function as key regulators in a variety of biological processes, exerting a significant impact on both the progression of normal development and the manifestation of various health and disease states^15^. The dysregulation of these small molecules emerges as a critical factor influencing cancer hallmarks. Thus, underscoring their involvement in oncogenesis, miRNAs are instrumental in modulating essential cellular functions, including evading growth suppressors, sustaining proliferative signaling, generating angiogenesis, activating invasion and metastasis, and resisting apoptosis^48,49^. The involvement of miRNAs in cancer is manifested through their engagement in post-transcriptional regulation, where they interact with their target genes by binding to complementary target mRNAs^50^. MiRNAs expression profile is progressively gaining prominence as one of the most promising biomarkers for diagnosing and treating cancers, including HNSCC^51,52^. Several miRNAs are used in HNSCC diagnosis by influencing oncogenic mechanisms. For instance, microRNA-125a promotes cancer cell proliferation and migration through the suppression of p53 protein expression, while microRNA-134 contributes to tumorigenesis and metastasis by downregulating the WWOX gene^53^. As a result, identifying miRNA targets is critical for gaining a full comprehension of the complexities of miRNA-mediated regulatory pathways and improving our understanding of the molecular mechanisms behind cancer development. As a tumor regulator, miR-103a-3p can control tumor progression in various cancers^21,25–56^. To the finest of our understanding, this research represents the initial report on the prognostic significance of hsa-miR-103a-3p as an oncogenic miRNA in HNSCC.

In the present study, by focusing on an underexplored miRNA, we aim to evaluate the potential of hsa-miR-103a-3p as a diagnostic biomarker. It is essential to clarify its primary molecular mechanisms and functions in cancer through various bioinformatics analyses. Accordingly, first, the examination of hsa-miR-103a-3p expression status yielded significant results. These findings indicated alterations in the regulation of hsa-miR-103a-3p, specifically showing elevated expression levels in HNSCC tissue samples when compared to healthy controls. This significant increased expression level in malignant tissues supports the active involvement of hsa-miR-103a-3p in the development of HNSCC, underlining its role as a major factor in disease progression. Validating the potential oncogenic role of the significantly upregulated hsa-miR-103a-3p profile, existing reports highlight its association with the development of various cancers. As it has been implicated in the progression of non-small cell lung cancer by influencing the *AKT* pathway through the targeting of *PTEN*^57^. Additionally, its involvement in gastric cancer has been linked to the silence of the *ATF7* target gene^25^. These findings align with and affirm the validity of our results. Consequently, the identified hsa-miR-103a-3p may serve as a valuable biomarker, offering potential utility in predicting outcomes for HNSCC patients. Considering, its application extends beyond, serving as a diagnostic tool for various types of cancer that have already been investigated^21,25–55,57,58^.

As for the prognostic significance of hsa-miR-103a-3p among patients diagnosed with HNSCC. The findings of our study proved a significant relationship between the abundance of hsa-miR-103a-3p in cancerous tissue samples, and decreased survival rates of HNSCC. Furthermore, this observed correlation between the highly expressed hsa-miR-103a-3p and poor overall survival mirrored comparable findings identified in several cancer types, including cervical, breast, and colorectal cancer^54,55,59^. Therefore, the convergence of our data with prior research not only reinforces the reliability of our findings but also highlights its potential as a robust and versatile prognostic indicator.

MiRNAs regulate a variety of cellular processes by binding to the 3’ untranslated region (3’UTR) sequences of target genes^60^. To improve the accuracy of has-miR-103a-3p predictions, the selected targets were refined further by sorting them according to the accessibility of binding sites. As a result, *UBE2Q1*, *ZFPM2*, and *ATXN1* were identified as highly ranked genes for hsa-miR-103a-3p in the list. *UBE2Q1*, a potential member of the ubiquitin-conjugating enzyme family (E2), has previously been linked to clinicopathological characteristics and patient survival in HNSCC^61^. Similarly, *ZFPM2-AS1*, *ZFPM2* potential target and an oncogenic long non-coding RNA (lncRNA), has revealed its prognostic importance in the context of HNSCC^62^. Furthermore, *ATXN1* has been found to be significantly hypomethylated in HNSCC tissues as compared to healthy tissues^63^. In addition, these genes exhibited significant alterations in other cancer types, suggesting their involvement in carcinogenesis. For instance, *UBE2Q1* has been observed to undergo mutation in colorectal cancer^64^ and breast cancer^65^ by suppressing the activity of the P53 gene. Furthermore, elevated expression of *ZFPM2* was observed in malignant pleural mesothelioma tissues compared to normal samples^66^, and the genetic alterations in *ZFPM2* may be associated with a poorer prognosis in hepatocellular carcinoma^67^. The suppression of ATXN1 regulation leads to the induction of epithelial–mesenchymal transition (EMT) in cervical cancer cells. These findings underscore the many-sided role of these genes in various cancer types and emphasize its significance in influencing critical molecular pathways. Moreover, the 18 significant DEGs indicated abundant expression levels in *HOXD10*, *HMGA2*, and *THY1*, whereas diminished expression was observed in *PDK4*, *AMOT*, and *UBL3*. According to F. Hakamy et al., the expression levels of *HOXD10* in HNSCC vary with cancer stage, whereby higher levels are linked to enhanced cancer cell proliferation, while lower expression facilitates metastasis and invasion^68^. Behzad Mansoori et al. reported the *HMGA2* plays a crucial regulatory role in cancer development. It is involved in inducing cancer cell proliferation, inhibiting cancer cell apoptosis, promoting metastasis, and contributing to cancer drug resistance^69^. Furthermore, elevated expression has been associated with a variety of malignancies, including HNSCC^70^. Additionally, it was documented that the expression level of *THY1* is elevated in HNSCC tissue compared to its corresponding normal tissue^71^. On the other hand, Young-Suk Jung reported that, through RT-PCR analysis, *PDK4* exhibited higher expression in HPV negative cells compared to HPV positive cells^72^. Furthermore, the *PCAT19*/miR-182 axis may influence *PDK4* function in laryngocarcinoma cells, impacting cell proliferation via altering mitochondrial metabolism and glycolysis^73^. *AMOT-p80*, an AMOT isoform, role in HNSCC is dependent on its level of expression: low expression is related with functions in metastasis and invasion, whereas high expression relates to proliferation and migration. Furthermore, AMOTs, which are involved in the Hippo-*YAP1* pathway, have been found to have lower expression levels in HNSCC^68,74^ *UBL3* has been discovered as a tumor suppressor in non-small cell lung cancer, implying its significance in preventing carcinogenesis, and as previously stated, hsa-miR-103a-3p has an oncogenic effect in this cancer type^57,75^.

The next step involved an in-depth analysis of the target genes of hsa-miR103a-3p. The findings uncovered significant associations between the target genes and key biological pathways, highlighting correlations with proteoglycans in cancer, the *cAMP* signaling pathway, miRNAs in cancer, and leucocyte transendothelial migration. Proteoglycans, integral components of the extracellular matrix, play diverse roles in cancer and angiogenesis. As highlighted by Yun Zhu et al., proteoglycans demonstrate diagnostic and prognostic significance in esophageal squamous cell carcinoma. Moreover, they can modulate the migration and invasion of esophageal cancer cells^76^. Yasutsugu’s resaerch team claimed that controlling intracellular *cAMP* levels through epinephrine inhibits the invasive potential of oral squamous carcinoma cells^77^. In addition, Numerous studies have shed light on the role of miRNAs in HNSCC, highlighting their potential utility as diagnostic biomarkers. MiRNAs that fall under this category include, but are not limited to, miR-21, miR-96-5p, miR-134, miR-361-3p, miR-101, etc^52,78–81^. Exosomes enriched with miR-9 derived from HPV-positive HNSCC have been shown to drive macrophage polarization toward the M1 phenotype, thereby enhancing the radiosensitivity of HPV-positive HNSCC cells^82^. As tumor cells stimulate a stromal response crucial for their proliferation and metastasis, Heena Zainab et al. have conveyed in their findings that desmoplastic reactions may serve as a prognostic biomarker in the context of oral squamous cell carcinoma^83^. Hence, the outcomes derived from the functional enrichment analysis align seamlessly with the findings presented in our study and underscore the coherence and validity of our research. Furthermore, GO biological processes related to the increase of the RNA metabolic process and the positive regulation of macromolecule biosynthetic processes were found to be enriched. These biological processes involve activities that increase the occurrence and rate of chemical reactions and pathways involving macromolecules. In terms of disease ontology, it was noted that the common target genes play a role in a various type of malignancies, including liver cancer, skin cancer, and breast cancer, among others. As a result, the results derived from the functional enrichment analysis are consistent with the findings of our research, suggesting that it could be possible for hsa-miR-103a-3p to have oncogenic influence on HNSCC.

Therapeutic modulation of microRNAs has emerged as a promising strategy in cancer treatment. This can be achieved either through miRNA mimics, which restore or enhance the activity of downregulated tumor-suppressive miRNAs, or through miRNA inhibitors (antagomiRs), which suppress the function of oncogenic miRNAs^84^. Advances in smart nanocarrier systems have further enhanced the precision and efficiency of miRNA-based therapies by enabling targeted delivery and reducing off-target effects. Such approaches allow selective modulation of key oncogenic signaling pathways that drive tumor progression in pancreatic, breast, and lung cancers^85^. Importantly, several preclinical studies have demonstrated that combining miRNA therapeutics with conventional chemotherapeutic agents produces synergistic anti-tumor effects, resulting in significantly improved treatment efficacy compared with either therapy alone^86^. These findings underscore the potential of miRNA-based interventions as both standalone therapeutic agents and powerful adjuvants to existing treatment regimens, paving the way for their translation into clinical applications.

## Conclusion

**Fig. 5:**
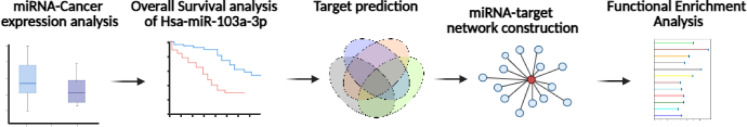
Bioinformatics analysis to explore the potential prognostic utility of hsa-miR-103a-3p in HNSCC.

In brief, our data highlights the diagnostic and prognostic importance of hsa-miR-103-3p in HNSCC. The upregulation of this miRNA was found to be oncogenic in HNSCC tissue when compared to healthy controls. Furthermore, the KM plotter online database consistently confirms the link between higher hsa-miR-103a-3p levels and a reduced survival duration for HNSCC patients. We identified 297 targets for hsa-miR-103a-3p using several bioinformatics methods and target prediction techniques, demonstrating enrichment in many processes related with carcinogenesis such as cAMP signaling pathway and proteoglycans in cancer pathway (Fig. 5).

## Limitations of the study

While this study provides novel insights into the prognostic potential of hsa-miR-103a-3p in HNSCC, several limitations should be acknowledged. The reliance on publicly available datasets introduces heterogeneity across patient populations, including variations in HPV status, tumor stage, treatment history, and sequencing platforms, which may affect reproducibility and generalizability. Additionally, inconsistencies in normalization methods and small sample sizes in some datasets further contribute to variability in expression profiles. Although the bioinformatics analyses highlight strong associations between hsa-miR-103a-3p, target genes, and cancer-related pathways, the study is limited by its in-silico nature. Experimental validation using in vitro and in vivo models is essential to confirm the functional role of this miRNA, and stratified analyses (e.g., by HPV status or treatment response) will be critical to clarify its clinical utility. Future prospective studies with well-characterized cohorts are therefore needed to establish causality, validate prognostic robustness, and explore the potential of hsa-miR-103a-3p as a biomarker for personalized medicine.

## Supplementary Information

Below is the link to the electronic supplementary material.

Supplementary Material 1

## Data Availability

The datasets used or analysed during the current study available from the corresponding author on request.

## Abbreviations

HNSCC: Head and neck squamous cell carcinoma
miRNA (miR): MicroRNA
dbDEMC: Database of differentially expressed miRNAs in human Cancers
GEPIA2: Gene expression profiling of interactive analysis
GO: Gene ontology
GEO: Gene expression omnibus
KEGG: Kyoto Encyclopedia of Genes and Genomes
