# Supplementary material for: Bioinformatics analysis to explore the potential prognostic utility of hsa-miR-103a-3p in head and neck squamous cell carcinoma
Source: Sci Rep. 2025 Nov 21;15:41159. doi: 10.1038/s41598-025-26188-6 (PMC12639060; doi:10.1038/s41598-025-26188-6)
Supplement: Supplementary file 1 — Supplementary Material 1 [file 41598_2025_26188_MOESM1_ESM.docx]

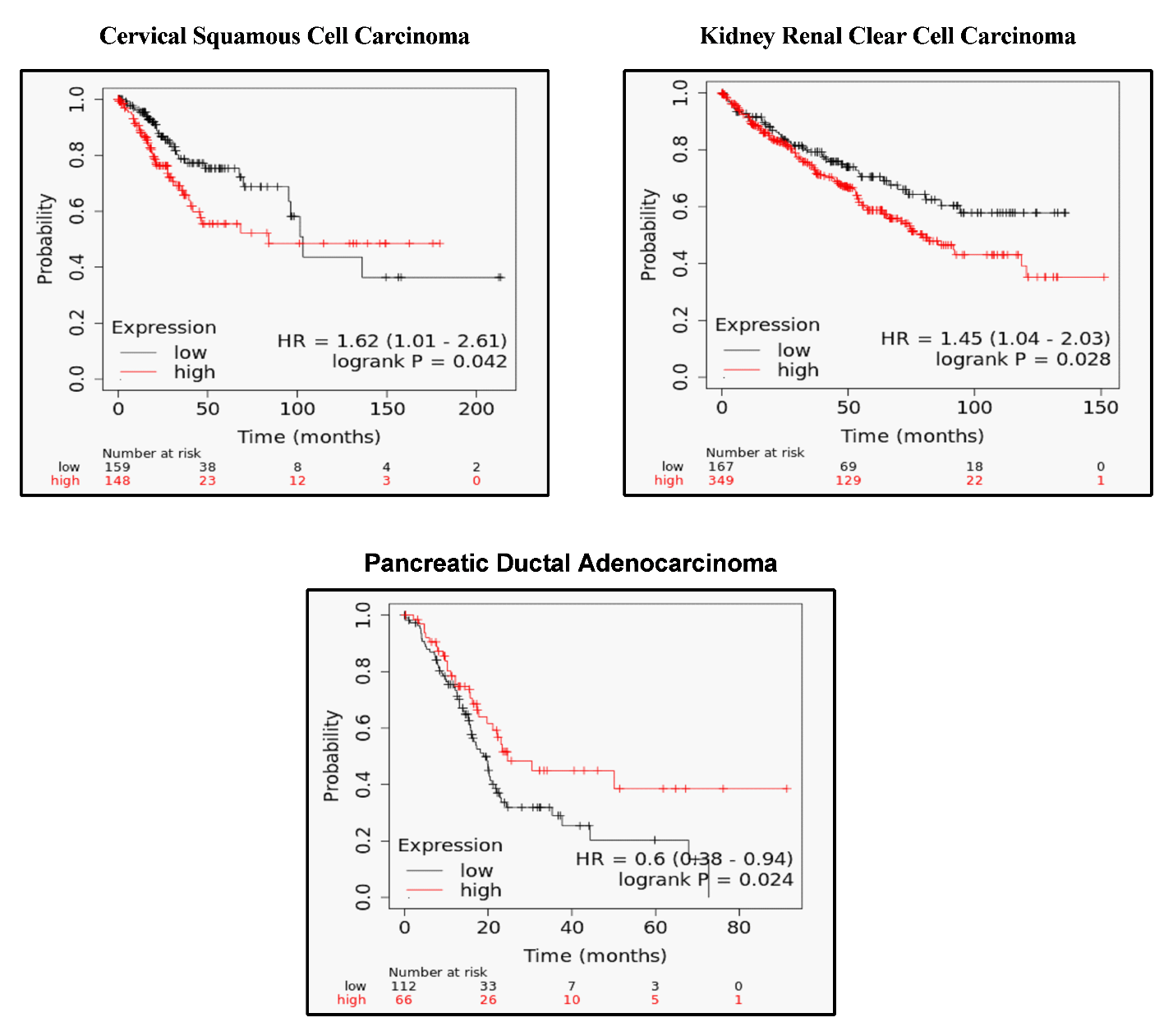
**Supplementary Information**

**Figure S1**. Survival analysis of hsa-miR-103a-3p in different cancer types


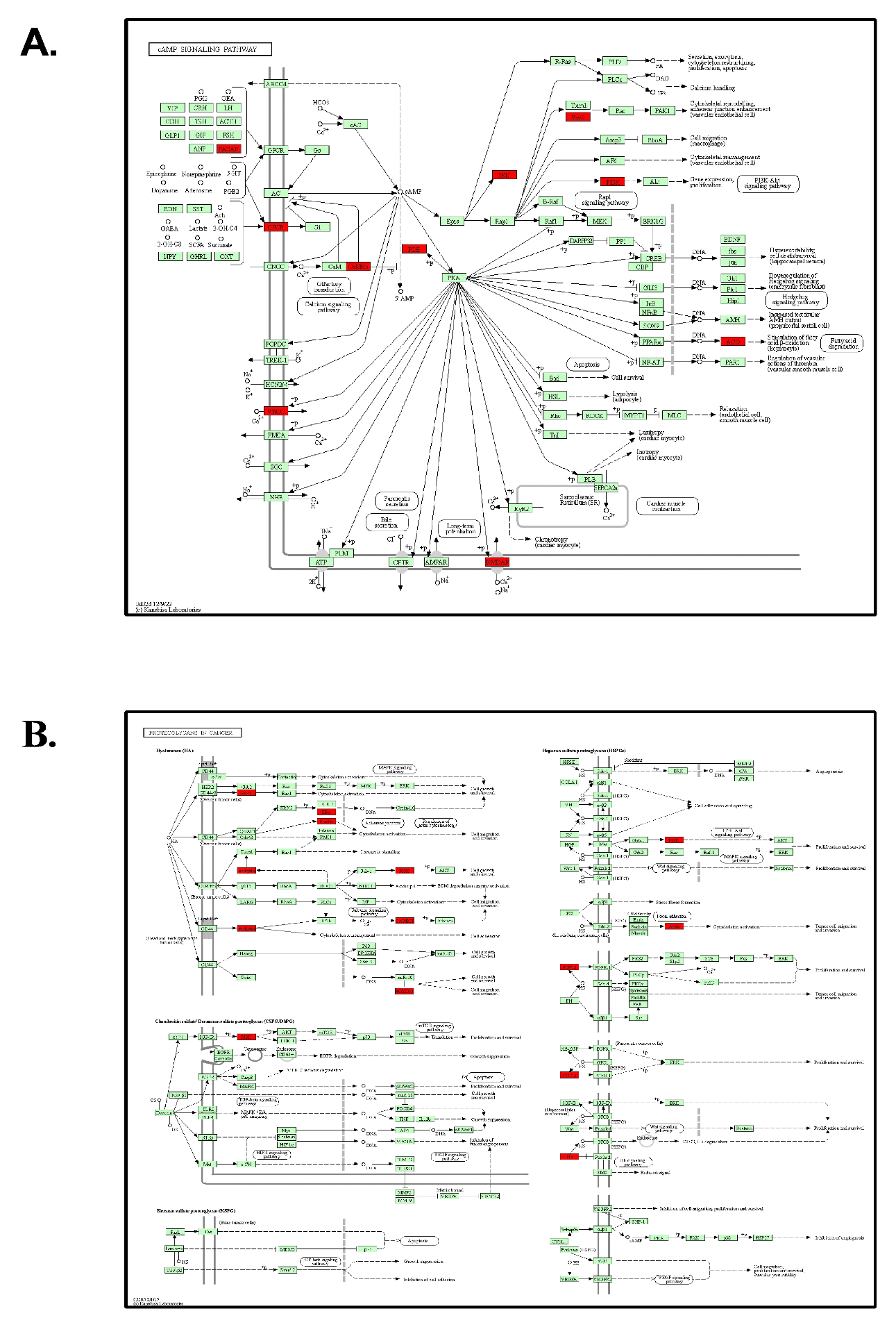


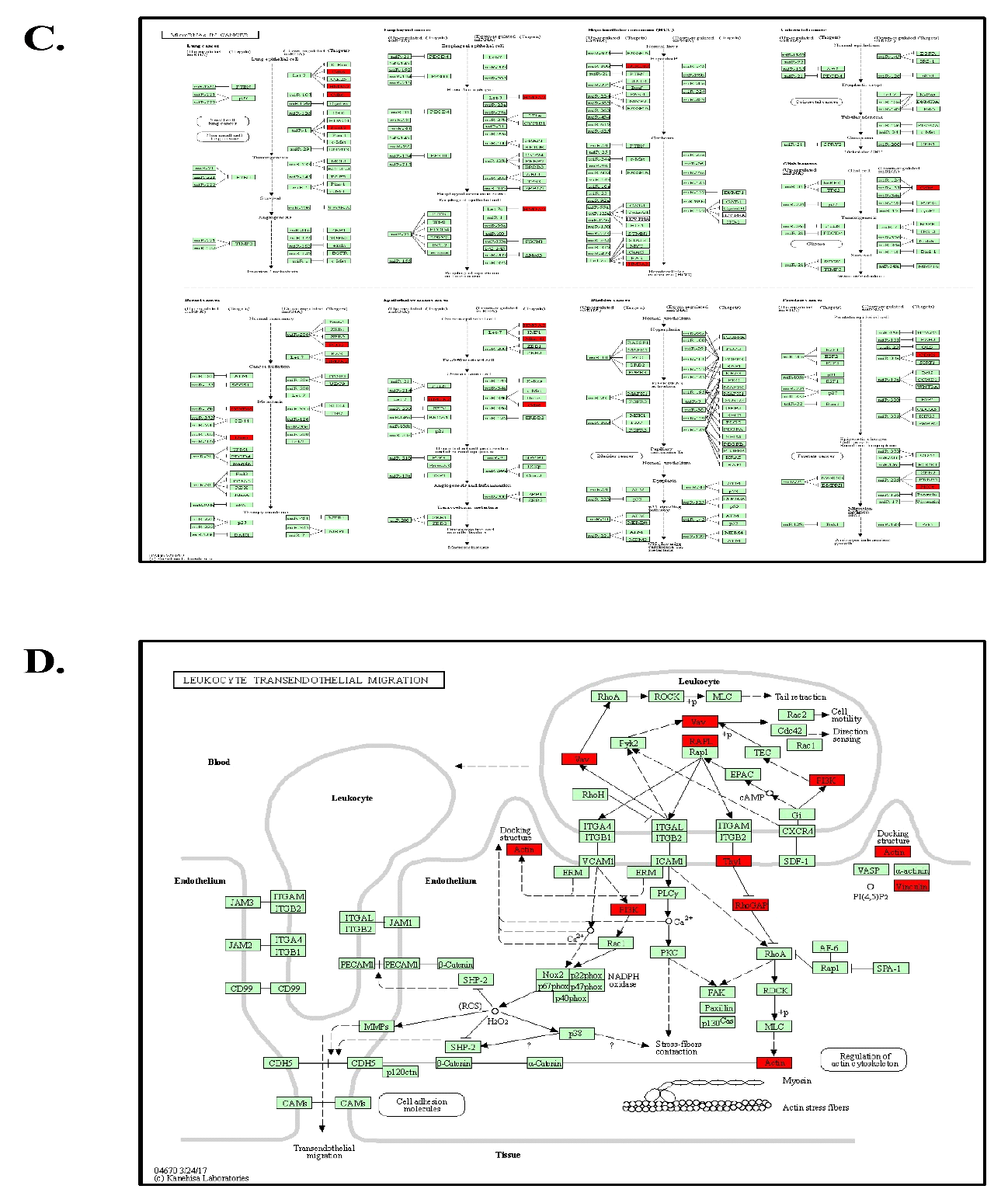


**Figure S2.** The highest enriched KEGG pathways. (A) cAMP signaling pathways (map04024) (B) proteoglycans in cancer (map05205) (C) miRNAs in cancer (map05206) (D) leukocyte transendothelial migration ([map04670](https://www.kegg.jp/entry/map04670)) ^1–3^


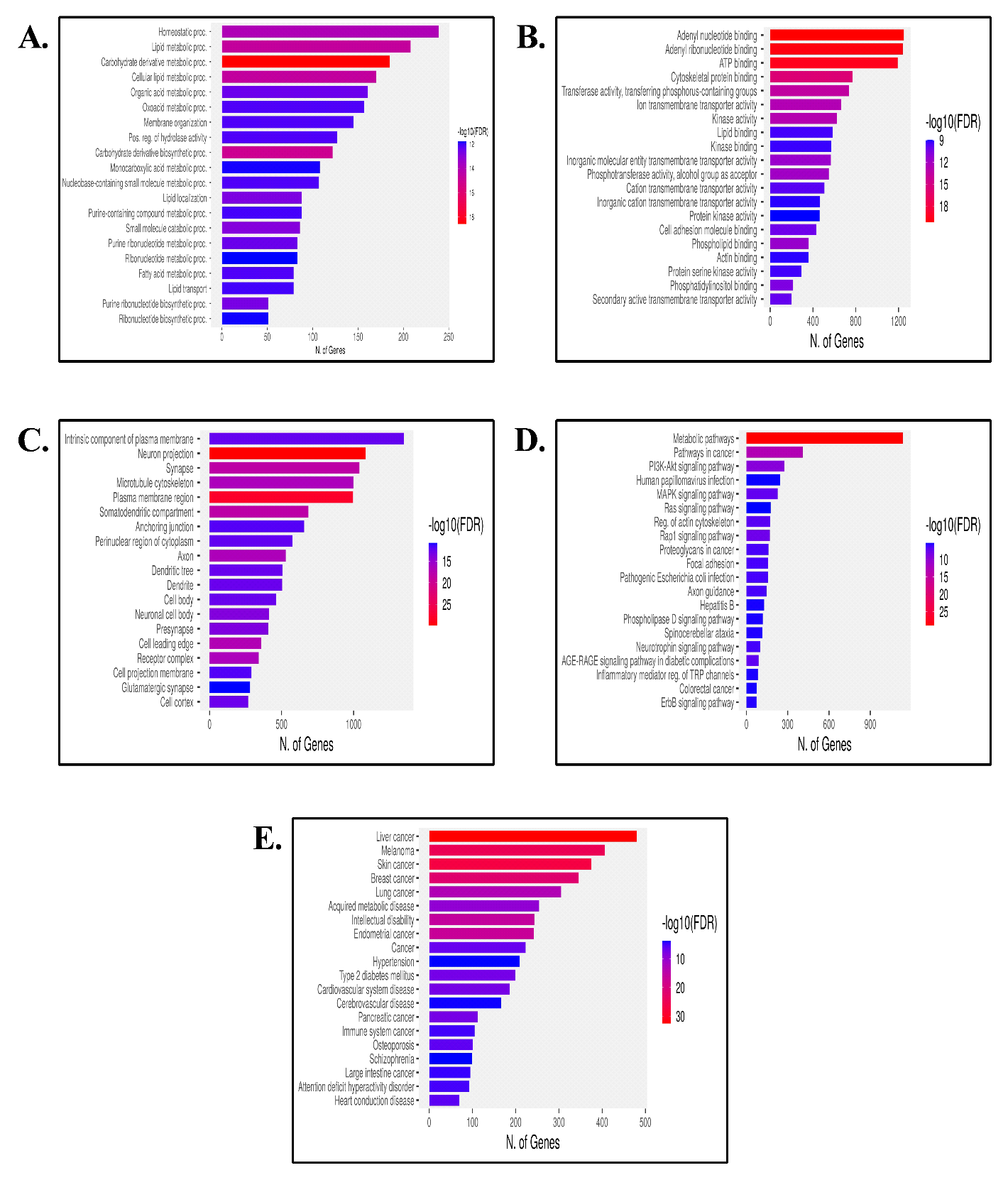


**Figure S3.** MiRwalk genes (with score higher than 0.95) GO analysis. (**A)** Biological Process. (**B)** Molecular Functions. (**C)** Cellular Components. (**D)** KEGG Pathways. (**E)** Disease Ontology.

**References**

1. Kanehisa, M., Furumichi, M., Sato, Y., Matsuura, Y., and Ishiguro-Watanabe, M. (2025). KEGG: biological systems database as a model of the real world. Nucleic Acids Res *53*, D672–D677. https://doi.org/10.1093/NAR/GKAE909.

2. Kanehisa, M., and Goto, S. (2000). KEGG: kyoto encyclopedia of genes and genomes. Nucleic Acids Res *28*, 27–30. https://doi.org/10.1093/NAR/28.1.27.

3. Kanehisa, M., Sato, Y., Kawashima, M., Furumichi, M., and Tanabe, M. (2016). KEGG as a reference resource for gene and protein annotation. Nucleic Acids Res *44*, D457–D462. https://doi.org/10.1093/NAR/GKV1070.
